# Reconstruction of porous media pore structure and simulation effect analysis of multi-index based on SNESIM algorithm

**DOI:** 10.1038/s41598-025-88730-w

**Published:** 2025-02-10

**Authors:** Qing Xie, Jiaqi Gao, Xiaochuang Ye, Jia Li, Yifei Song, Siwen Hu

**Affiliations:** 1 College of Petroleum Engineering, Xi’an Shiyou University, Xi’an, 710065 China; 2 MOE Engineering Research Center of Development & Management of Western Low &, Ultra-Low Permeability Oilfield, Xi’an, 710065 China; 3 NO.3 Gas Production Plant of Changqing Oilfield Company, Petro China, Xi’an, 710018 Shanxi China; 4 College of Electronic Engineering, Xi’an Shiyou University, Xi’an, 710065 China

**Keywords:** Coordination number, Variogram, Digital core, MPS, Porous media, Permeability, Energy science and technology, Mathematics and computing

## Abstract

The pore structure of porous media directly affects its permeability characteristics and fluid flow properties, making the accurate reconstruction of these structures of great significance. In recent years, multi-point statistics (MPS) methods have been widely used in pore structure modeling. Among them, the SNESIM algorithm, as an advanced MPS technique, has been extensively applied in the study of porous media pore structures. This paper aims to investigate the use of the SNESIM algorithm for reconstructing pore structures on 2D core slices with varying porosities, all taken from the same core. It also analyzes the effectiveness, limitations, and applicable conditions of the algorithm. This study utilizes CT scan images to construct digital core technology and applies the SNESIM algorithm to reconstruct pore structures of core slices with different porosities. By analyzing performance parameters such as porosity, pore throat ratio, average grain radius, coordination number, and permeability, the study shows that the reconstructed images(RI) from most samples maintain a trend similar to that of the training images(TI), demonstrating the good applicability and reliability of the SNESIM algorithm in pore structure reconstruction. However, the core slices used in this study were all taken from the same core. Effectively transferring the pore structures from the 2D plane to the 3D pore space and restoring the pore structures to the greatest extent still requires further research. In particular, when dealing with complex pore structures, the accuracy and performance of the SNESIM algorithm need further improvement. Future research will focus on optimizing the algorithm to handle more diverse pore structures and exploring 3D reconstruction methods to more comprehensively describe and analyze the pore characteristics in actual porous media.

## Introduction

In geological and geophysical research, MPS have garnered significant attention due to their superiority in modeling and analyzing complex geological structures. Porous media, as an important natural and artificial material, have extensive applications in fields such as petroleum and natural gas extraction, water resource management, and environmental engineering. However, the internal pore structure of porous media is complex, and accurately predicting fluid flow behavior within them is challenging^1,2^. Permeability, as a key parameter describing fluid flow characteristics in porous media, is crucial for accurately predicting fluid behavior within these materials. Therefore, accurately reconstructing the pore space structure of porous media to enhance the analysis of permeability and other pore performance parameters has become a major focus of current research.

The main methods currently used to study permeability include the Finite Difference Method (FDM)^3^, Pore-morphology Modeling^4^, Effective Medium Theory^5^, Computational Fluid Dynamics (CFD)^6^, and the Lattice Boltzmann Method (LBM)^7^. The LBM is an effective method for calculating the permeability of the microstructure of porous media. Siyu Chen (2019) used the LBM method to simulate fluid flow in porous materials and predict their permeability coefficients^8^. Z. Irayani (2018) established three vertical networks, combining the renormalization group method with LBM to calculate the permeability of 3D computed tomography rock images.^9^ Budi Dharmala Saputra (2024) studied the effect of coordination number on permeability in a 3D rock model using the LBM method, confirming that LBM can serve as a powerful tool for understanding pore-scale seepage^10^. In the study of porous media, permeability, as a key transport property, is closely related to parameters such as porosity. However, in addition to porosity, other geometric and topological parameters, such as tortuosity, Euler number density, and constriction, have also become crucial for a comprehensive understanding of the transport properties of porous media. Euler number density, as a simple stereological method, has been widely applied to estimate the connectivity of porous media^11^. Arns et al.(2000) further explored the evolution of microstructures with density using statistical measures based on Euler-Poincaré characteristics^12^. Moreaud et al.(2008) proposed a pore-to-pore tortuosity map based on geodesic constrained distance propagation for analyzing pore connectivity, enabling global quantification of the connectivity between pores^13^. Additionally, research by Bini et al.(2019) and Chaniot et al.(2024) has shown that tortuosity has a significant impact on diffusion rates and transport properties^14,15^. Furthermore, the A-protocol framework proposed by Chaniot et al. (2022), which combines local shape features with global topological structures, provides a new approach for analyzing permeability in porous media, offering additional insights into permeability research^16^.

Although MPS have made significant progress in reproducing features of complex geological reservoirs, traditional two-point geostatistics based on variograms are limited when dealing with large amounts of complex reservoir data. The pore structure reconstruction model is one of the most commonly used methods for pore reconstruction. This model primarily utilizes numerical simulation or computer graphics techniques to digitize the pore space, thereby reproducing the microscopic pores and framework structures of porous media. Presently, the main methods for reconstructing pore structures include the Discrete Element Method (DEM)^17,18^ and the Image Sequence Reconstruction Method^19,20^. The Discrete Element Method (DEM) refers to simulating particle size, pore radius, porosity, and other parameters through a series of particle accumulations to reflect the actual pore structure^21^. However, because DEM typically uses spherical particles to simulate the real reservoir structure, in the absence of realistic shape simulations of the pore structure, this reconstruction method cannot accurately reflect the complex pore morphology and structure of actual rocks^22,23^. In contrast, the Image Sequence Reconstruction Method leverages digital technology to reconstruct microscopic pore structures, resulting in a closer approximation to the actual pore structure. Thus, it is widely applied in pore reconstruction research. This method uses two-dimensional scanned images of porous media samples obtained through digital imaging technology and applies these 2D images to pore structure modeling using computer graphics^24,25^. Currently, commonly used imaging methods mainly include Computerized Tomography (CT) imaging technology^26^, Scanning Electron Microscopy (SEM)^27,28^, and Focused Ion Beam/Scanning Electron Microscopy (FIB/SEM) imaging technology^29^. Among these, CT scanning technology is relatively mature and widely used^30–32^. Using CT scan images as prior data for reconstruction images, combined with multipoint statistical methods, is a current research hotspot^33,34^. Biswal adopted a physics-based approach to simulate the structure of porous media^35^. Julien Straubhaar proposed a method for editing static images to generate the features of training images while maintaining the consistency of the overall spatial structure^36^.

To overcome these limitations, Strebelle and Journel (2000) proposed the single normal equation, which estimates the data distribution of unknown nodes by calculating the probability of node occurrence in training images^37^. However, this method relies on an iterative approach, and the computation speed directly affects the speed of pore structure construction. Strebelle (2002) developed a data storage method based on a search tree, called the Single normal equation simulation (SNESIM) algorithm. By scanning the training image once, the obtained multipoint probabilities are stored in the “search tree” nodes^38^. Therefore, MPS is a pixel-based direct sampling algorithm. This method first assigns conditional data values as initial data in the simulation grid and then fills the data values of unknown grids in the training image in a random order^39–41^. Julien Straubhaar (2021) extended the direct sampling algorithm using MPS tools to address unequal data^42^. Xiaoqi Zhou (2023) simulated the heterogeneity and spatial trends of subsurface formations using a knowledge-based MPS and combined it with standard permeability test data to improve simulation accuracy^43,44^. To optimize the computational cost of SNESIM while ensuring the quality of pattern reproduction, Strebelle et al.(2014) proposed a way to minimizes the computational burden by adjusting the size of the data template. In this approach, the minimum acceptable template size is selected by setting a threshold; beyond this threshold, additional conditional data do not improve the estimation of conditional probabilities^45^. In addition, Senyou An et al. (2020) studied a pore network model based on Graphics Processing Unit (GPU) is developed to simulate flow and transmission in a network with millions of holes, which greatly improves the operating rate^46^. However, when the search tree fails to find similar data events that match the estimated point and conditional data, Johannsson et al. (2021) proposes to use the the global probability from training image is used instead of the local conditional probability, leading to a decrease in estimation accuracy^47^. Therefore, the SNESIM algorithm still faces certain challenges, such as sensitivity to template size and shape, the complexity of simulation grid allocation, and high computational costs when handling high-dimensional complex structures. These factors directly affect the accuracy of reconstructed pore structures and the reliability of permeability calculations, limiting its application in real-world complex geological environments.

This study aims to utilize the SNESIM reconstruction algorithm by controlling parameters such as template size and grid quantity, using core slice samples of porous media with varying porosity as prior data to generate corresponding pore structure images. By analyzing differences in parameters such as porosity, average pore diameter, pore granularity, pore coordination number, and permeability, the study explores methods to improve reconstruction accuracy. The contribution of this research lies in providing new insights into the application of SNESIM in reconstructing porous media structures and laying the foundation for further enhancements in model performance and accuracy.

## Methodology

The main idea of MPS method consists of three steps: conditional data extraction, feature library construction, and probabilistic simulation. First, real data obtained from geological reservoirs are used as conditional data to extract the actual image structural features of the geological reservoir. These extracted image features are stored using a “search tree” structure, forming a library of TI features, search tree is a hierarchical data structure used to organize and store the extracted data events from the TI. Finally, when generating simulated images, the relevant image features are selected from the feature library based on the conditional data and probabilistic principles, creating a reconstructed dataset that resembles the real geological structure.

$Z(n)$ is a spatial structural variable defined over the domain of the TI and the red represents the pores and the blue represents the skeleton. The data event $d(u)_{n}$ is the state value of size $n$ at the center location $u$. The data template $\tau_{{\text{n}}}$ includes a geometric pattern composed of $n$ vectors,$\tau_{n} = \{ h_{\alpha } ;\alpha = 1,2,...,n\}$, with the template center location set as $u$ and other template positions as $T$, $T = \{ u + h_{1} ,u + h_{2,} ...,u + h_{\alpha } \left( {\alpha = 1,2,...,n} \right)\}$_._

**Fig.1:**
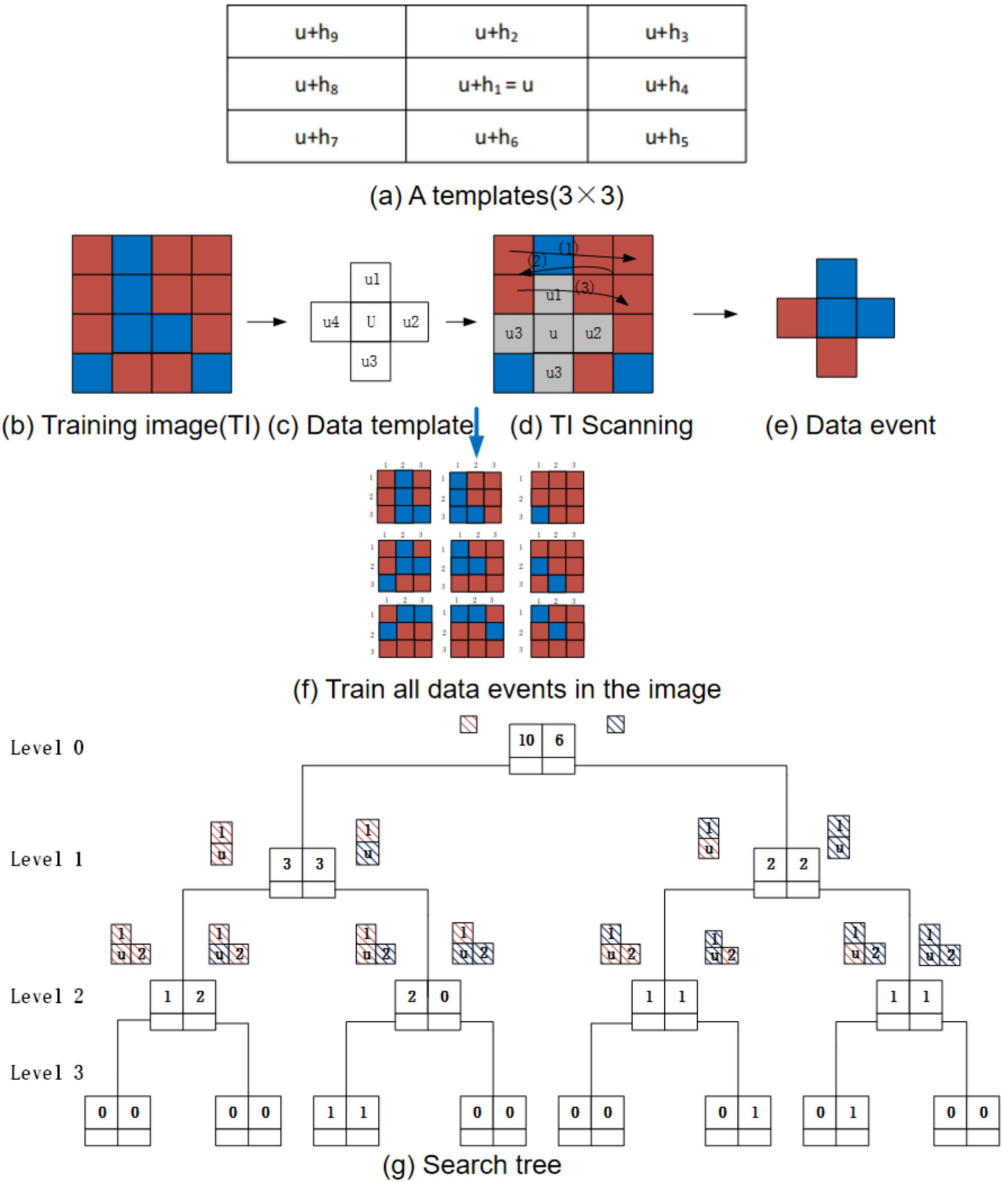
The process of scanning training images to generate data events to build search trees. (**a**) (A template(3 × 3)) shows a 3 × 3 data template $T$ represented by vectors, where $u$ indicates the central position of the template,$h_{1} = 0$, and $\alpha$ represents the number of nodes in the template. (**b**) (Training image(TI)) the grid template is composed of 4 × 4 pixels, determined by the center point $u$ and 15 vectors, with each vector represented by a grid point. (**c**) (Data template) shows the data template for a 2D data event with $n = 4$. (**d**) (TI scanning) illustrates the scanning of the TI using the data template in the direction indicated by the arrow to construct the search tree. (**e**) (Data event) Scanning the TI to obtain data events using a 3 × 3 template. (**f**) is a data event related to the data template. It illustrates the process of scanning the TI to obtain a data event. (**g**) the search tree constructed from training data events, showing hierarchical relationships among data templates. (**b**)-(**f**) illustrates the process of scanning the TI to obtain a data event. (red represents the pores and blue represents the skeleton).

Figure 1a shows a 3 × 3 data template $T$ represented by vectors, where $u$ indicates the central position of the template,$h_{1} = 0$, and $\alpha$ represents the number of nodes in the template. Typically, the data template is simplified to the form shown in Fig. 1c when scanning the TI. Figure 1b, TI is the initial image used for model training, containing known microstructural information of the porous medium, serving as the Search Tree. The grid template is composed of a 4 × 4 pixel grid, which is determined by a center point and 15 surrounding vectors, with each vector corresponding to a grid point. These vectors define the relative positions and orientations of neighboring pixels in the template, which is crucial for capturing the structural features of the TI. Figure 1c illustrates the data template used for scanning a 2D data event, where the template is applied to a specific region of the TI to extract relevant features. This data template helps in identifying and recording local structures within the image by capturing the relationships between pixels in the vicinity of the central point. Figure 1d depicts the process of scanning the TI using the data template. The scanning is carried out in the direction indicated by the arrow, and this step is essential for constructing the search tree. During the scanning, each position in the image is evaluated with respect to the template, which allows for the identification of matching regions. The search tree is built by sequentially recording the occurrences of similar data events, facilitating the reconstruction or analysis of the image. Figure 1e shows data events obtained by scanning TI using a 3 × 3 template. It illustrates an example of the data events generated when scanning TI using the data template shown in Fig. 1c. Specifically, the scanning process is carried out until the position indicated by the gray area in Fig. 1d is reached. The data event is extracted at this specific location, corresponding to a particular configuration of pixel values within the TI. A data event refers to a specific pixel configuration or region extracted from the TI, used to describe the microstructural features of the porous medium. Figure 1d specifically highlights a data event, which is the result of applying the data template to a region of the TI. It shows the detailed process of scanning and extracting data events from the image, which are then used to build the search tree and facilitate further analysis. Figure 1f shows all the data events generated by scanning the TI in Fig. 1b using the data template from Fig. 1c. In this step, the data template is systematically applied across the entire TI, capturing all the relevant local features that match the template structure. Each data event corresponds to a region of the TI where the template is found, and these events are recorded to provide a comprehensive representation of the image’s structural information. The result is a collection of data events that represent the local patterns or features identified throughout the image, which are then used for further analysis or reconstruction. Finally, Fig. 1g presents the search tree structure, which is generated from the scanning process. This tree organizes data events based on their similarities and relationships, facilitating efficient retrieval and analysis of relevant image features.

Under the assumption of stationarity, the ratio of the number of repetitions of a data event in the TI to the size of the TI is equivalent to the probability of occurrence of that data event. In the simulation, the state value of $Z(n)$ is determined by the conditional probability distribution function (cpdf ${\text{d}}\left( u \right){\text{ = \{ z(u}}_{\alpha } {\text{) = s}}_{{k_{\alpha } }} {;}\alpha { = 1,2}...{\text{,n\} }}$ extracted from the TI. According to Bayes’ conditional probability formula:

$$\Pr ob\left\{ {Z(u) = s_{k} |d({\text{n}})} \right\} = \frac{{c_{k} \left( {d\left( {\text{n}} \right)} \right)}}{{c\left( {d\left( {\text{n}} \right)} \right)}} \approx \frac{{c_{k} \left( {d\left( {\text{n}} \right)} \right)}}{{N_{n} }}$$

$c\left( {d\left( {\text{n}} \right)} \right)$ is the repetition count of $d(n)$ for the data event,$c_{k} \left( {d\left( {\text{n}} \right)} \right)$ is the inferred repetition count from $c\left( {d\left( {\text{n}} \right)} \right)$ when the central node $Z(u)$ has the value $Z(n)$. The specific explanations of certain symbols and parameters in the above formulas can be found in the Appendix.

Therefore, by scanning the TI, the conditional probability density function at unsampled points can be obtained. Using this conditional probability density function, random simulation is performed at the points to be simulated, until all the points have been simulated. Therefore, the probability of occurrence of the conditional data event can be converted to the ratio of the size of the 2D binary image of the effective initial pore $N_{n}$.

**Fig. 2:**
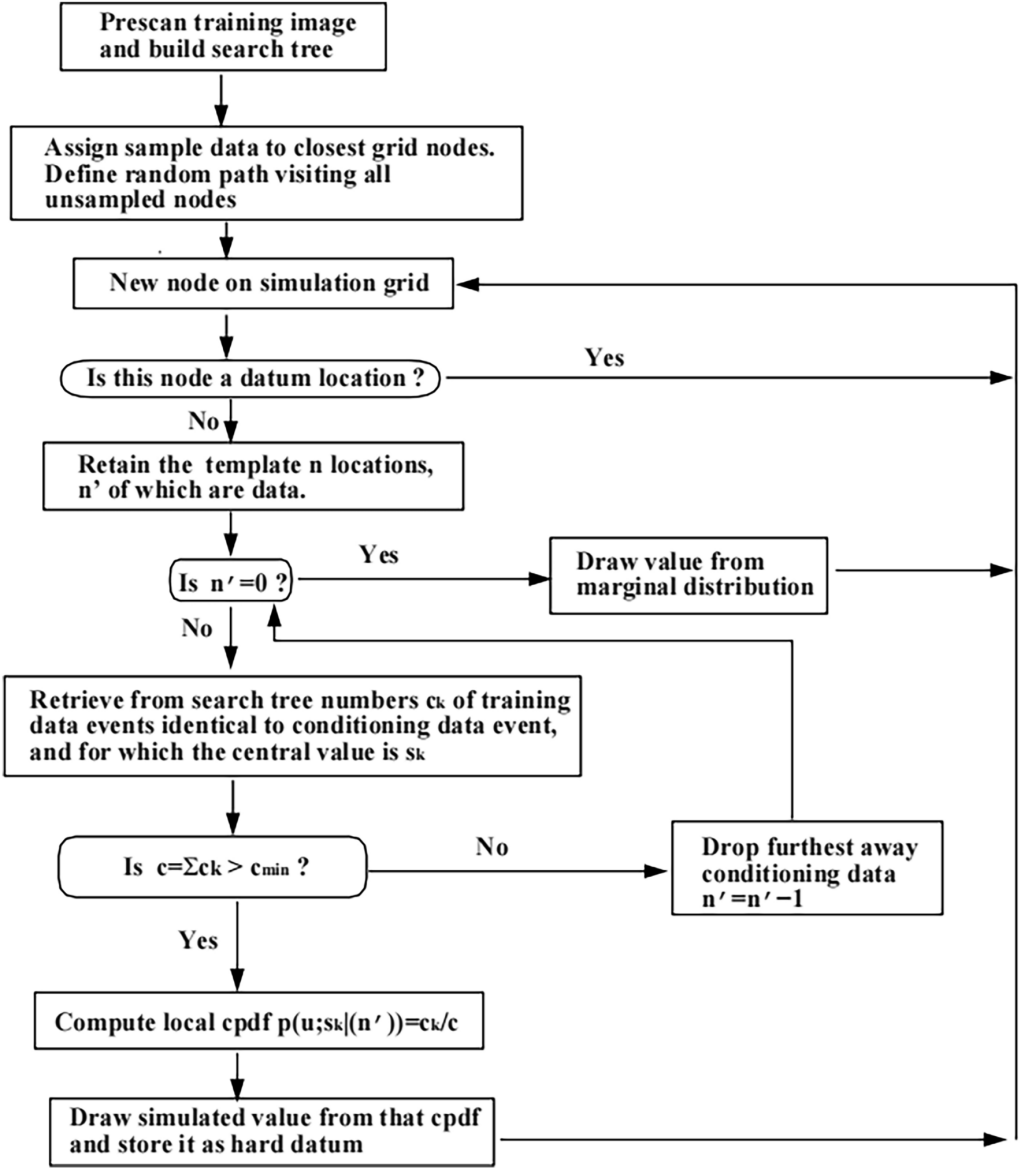
Flowchart of SNESIM algorithm. n = unsampled grid nodes; sk = conditional data event center value; k = number of conditional data events; p(u; sk | (n)) = conditional probability.

The main process of the SNESIM algorithm is as follows:

1. Pre-scan TI and Build the Search Tree: Assign sample data to the nearest grid nodes and define a random path to traverse all unsampled nodes. Check if the current node is on the simulation grid; if it is, continue; otherwise, move to the next node according to the random path. If the current node is a location with existing data, skip to the next node. Retain information about the positions in the template where n positions have existing data.
2. Check for Data Positions: Determine if there are any data positions (n' ≠ 0). If not, draw a value from the marginal distribution as the simulation value. Retrieve the number of training data events from the search tree that match the conditional data event, and obtain these events’ central values as $s_{k}$.
3. Verify Event Count: Check if the number of retrieved data events $c = \sum \alpha_{k}$ is greater than the minimum value $c_{\min }$. If not, remove the most distant conditional data and recalculate.
4. Calculate Local Conditional Probability Density Function: Compute the local conditional probability density function $$p\left( {u;sk(n^{\prime})} \right) = {\raise0.7ex\hbox{${\alpha_{k} }$} \!\mathord{\left/ {\vphantom {{\alpha_{k} } c}}\right.\kern-0pt} \!\lower0.7ex\hbox{$c$}}$$ for subsequent simulation value extraction.
5. Extract and Store Simulation Value: Draw a simulation value from the local conditional probability density function and store it as hard data.
6. Fig. 2 illustrates the flowchart of the SNESIM algorithm. SNESIM reconstruction algorithm is shown in the Table 1^37^.

**Table 1:** Algorithm: SNESIM (single normal equation simulation).

1. Define a data template ${\uptau }_{{\text{n}}}$
2. Pre-scan training image to store multiple-point patterns specific to ${\uptau }_{{\text{n}}}$
3. Relocate hard data to the nearest simulation grid node and freeze them
4. Define a random path visiting all locations to be simulated
5. for each location $u$ along the path do
6. Find the conditioning data event ${\text{d}}_{{\text{n}}}$ defined by template ${\uptau }_{{\text{n}}}$
7. Retrieve the conditional cumulative distribution function(CCDF) ${\text{Prob\{ Z(u) = s}}_{{\text{k}}} {\text{|d}}_{{\text{n}}} {\text{\} }}$ from patterns got in step 2
8. Draw a simulated value ${\text{z(u)}}$ from that conditional probability and add it to the data set
9. end for

### Application example

**Fig.3:**
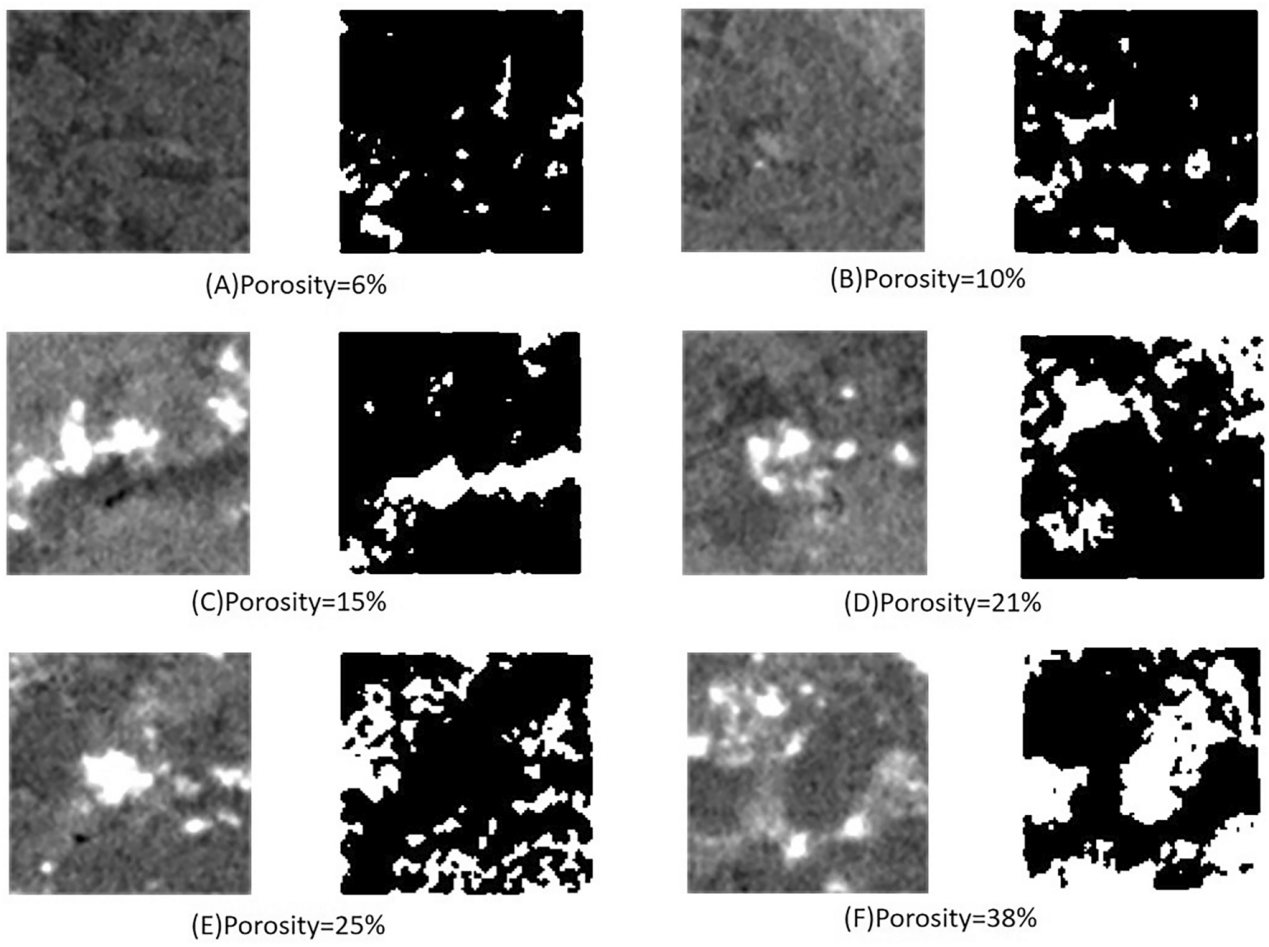
TI under different porosity levels: columns one and three represent grayscale images; columns two and four represent binary images (black for rock matrix, white for pores).

In this experiment, samples were selected from actual reservoir cores from an oil field. Six core slices with porosities of 6%, 10%, 15%, 21%, 25%, and 38% were extracted from the 3D scanned images of the cores, labeled sequentially as A-F, as shown in the first and third columns of Fig. 3 The second and fourth columns in the figure display the binarized images of the CT scan, where the black parts represent the rock matrix and the white parts indicate the pores.

**Fig. 4:**
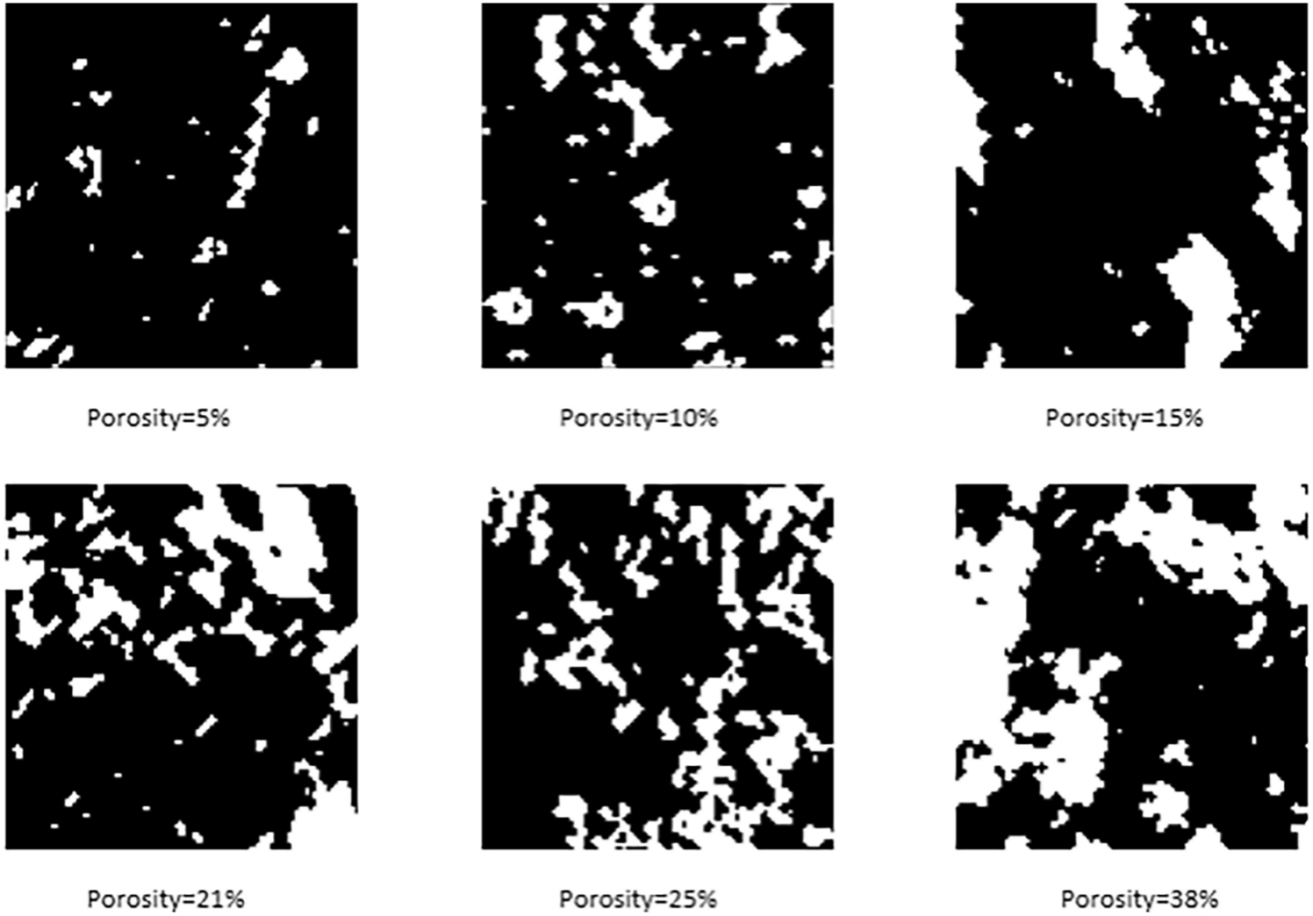
Reconstructed image (RI).

Figure 4 shows the RI of the six samples with porosities of 5%, 10%, 15%, 21%, 25%, and 38%. By comparing the pore structures of the initial TI with those of the RI, it can be observed that the RI retain the pore structure of the initial TI. Types of pore structures and heterogeneities, such as narrow pores, small pores, and large pores present in the initial images, are also reflected in the RI.

**Fig.5:**
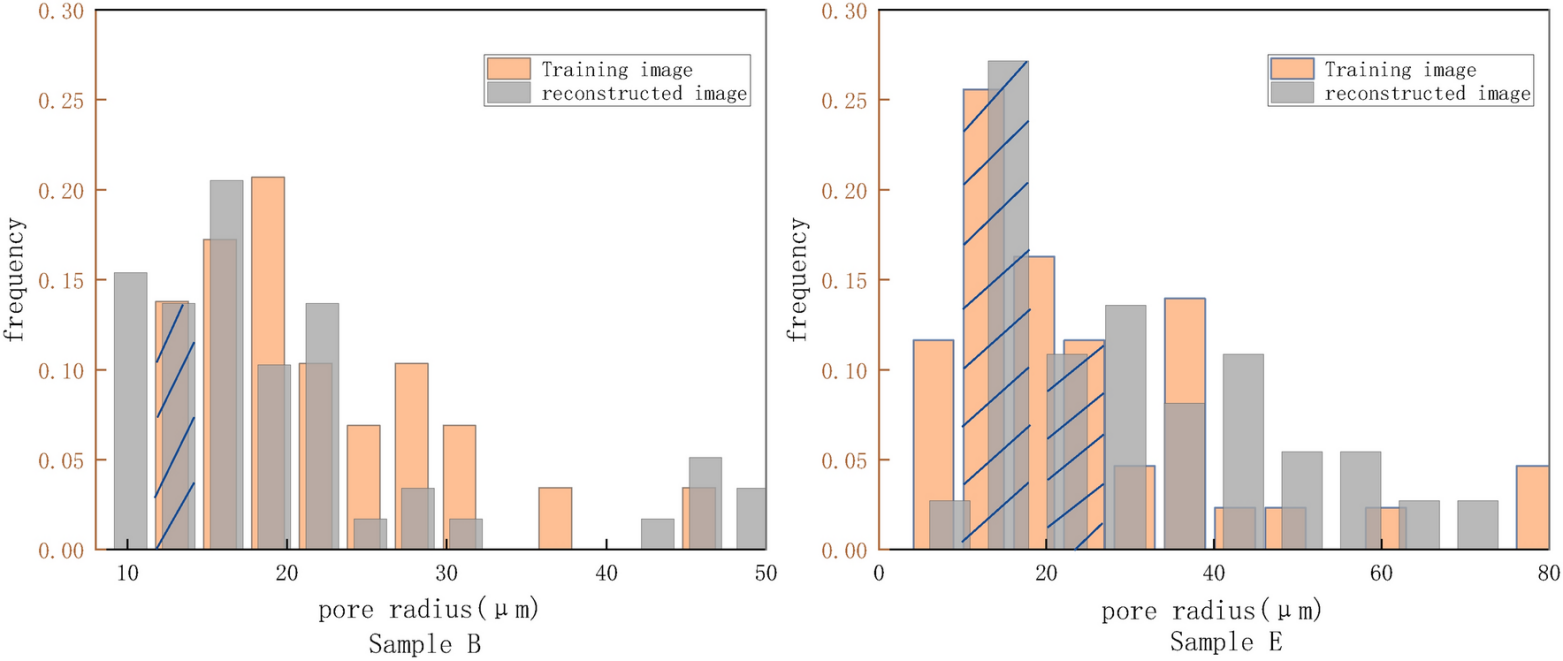
The pore size distribution for sample slices B and E (This figure shows the frequency of occurrence of different sample sizes in the pore images).

For example, in Fig. 5, samples B and E are illustrated. The bar charts display the frequency of different pore radii in the pore structure, with yellow bars representing the TI and gray bars representing the RI. Figure 5 attests of the similar trend of the pore radius and the occurrence frequency of the two samples. For instance, in sample E, the heights of the yellow and gray bars for pore radii of 15 μm and 21 μm, are quite similar, which is represented by the blue slash in the diagram.

**Fig. 6:**
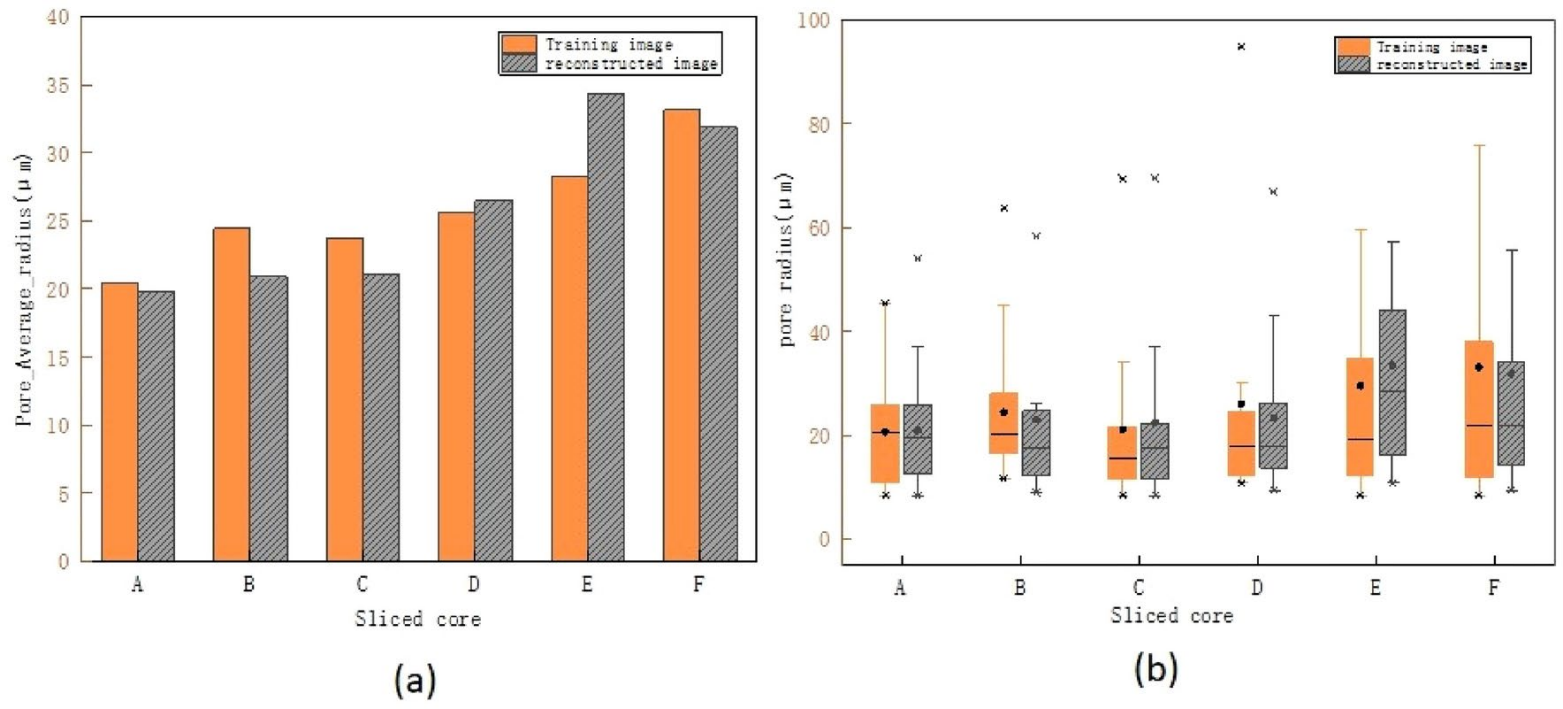
Impact of different pore radius distributions on the reconstruction effect of pore structures. (**a**) Average pore radius of the simulation realizations. (**b**) Pore diameter distributions of the simulation realizations.

In this paper, the "pore-throat ratio" index is used for quantitative analysis of the homogeneity within the pore structure of porous media. Within a local range, the variation between pores and throats changes with the pore-throat ratio; that is, the more pronounced the changes in pore structure at a fine scale, the poorer the homogeneity of the porous media. Figure 6 analyzes the reconstruction effects of pore structures at different porosities from the perspective of pore radius distribution. Figure 6a presents histograms of the average pore radius distribution for samples with different porosities. The yellow bars represent the average pore radius of the initial TI, while the gray bars represent the average pore radius of the RI. The bar charts show that the average pore radius of the RI generated by the algorithm is quite close to that of the initial TI, indicating good consistency between the two in this metric. Figure 6b shows boxplots of pore diameter distributions for samples with different porosities. The yellow and gray parts represent the initial TI and RI, respectively. Compared to the initial TI, the RI have a larger interquartile range (IQR) and wider upper and lower range edges, indicating higher variability in the RI. The median (average pore diameter) of the RI is slightly higher or comparable to that of the initial TI. The RI exhibit more frequent outliers, suggesting some deviation from the initial pore structure. Overall, the RI and initial TI are quite consistent in terms of median (average pore diameter) and general boxplot trends, demonstrating that the reconstruction algorithm is effective in capturing the average pore diameter. However, the increased variability (wider IQR and more outliers) in the RI may reflect the introduction of new features during the reconstruction process, indicating increased diversity.

**Fig. 7:**
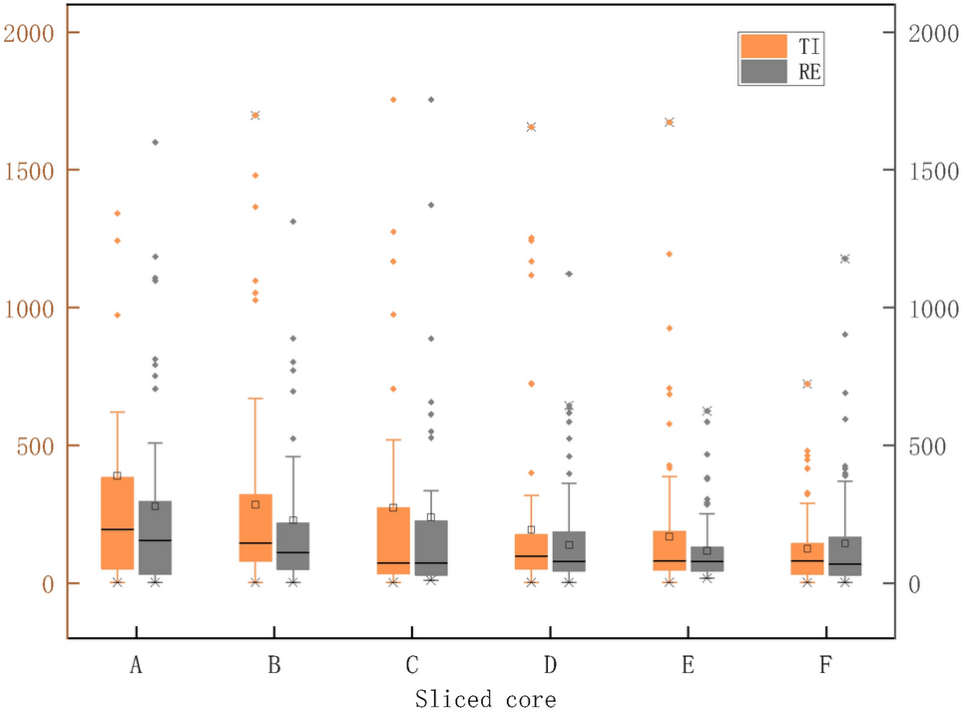
Pore-throat distribution of TI and RI for different slices (yellow represents TI, gray represents RI).

The yellow box in Fig. 7 is A box diagram of the average pore throat radius of six different porosity samples (A-F) in the initial TI. The gray boxes in Fig. 7 represent the box plots of the average pore-throat radius of the pore structures generated by the reconstruction algorithm for the same samples. Each box plot in Fig. 7 illustrates the distribution of the average pore-throat radius for a sample, including the quartiles, minimum, maximum, and outliers. Overall, the data distribution of the RI (yellow) is close to that of the TI (gray). The median and interquartile range (IQR) of the RI are roughly consistent with those of the TI, indicating that the average pore-throat radius of the RI is close to that of the initial pore structure. Although there may be a few outliers in some samples, the boxplots of the RI are almost identical in distribution to those of the TI, demonstrating that the reconstruction method performs well in preserving the pore-throat radius distribution characteristics. Particularly, the consistency in the median and IQR between the reconstructed and TI shows that the reconstruction method is successful in capturing and reproducing the main statistical features of the original data.

**Fig. 8:**
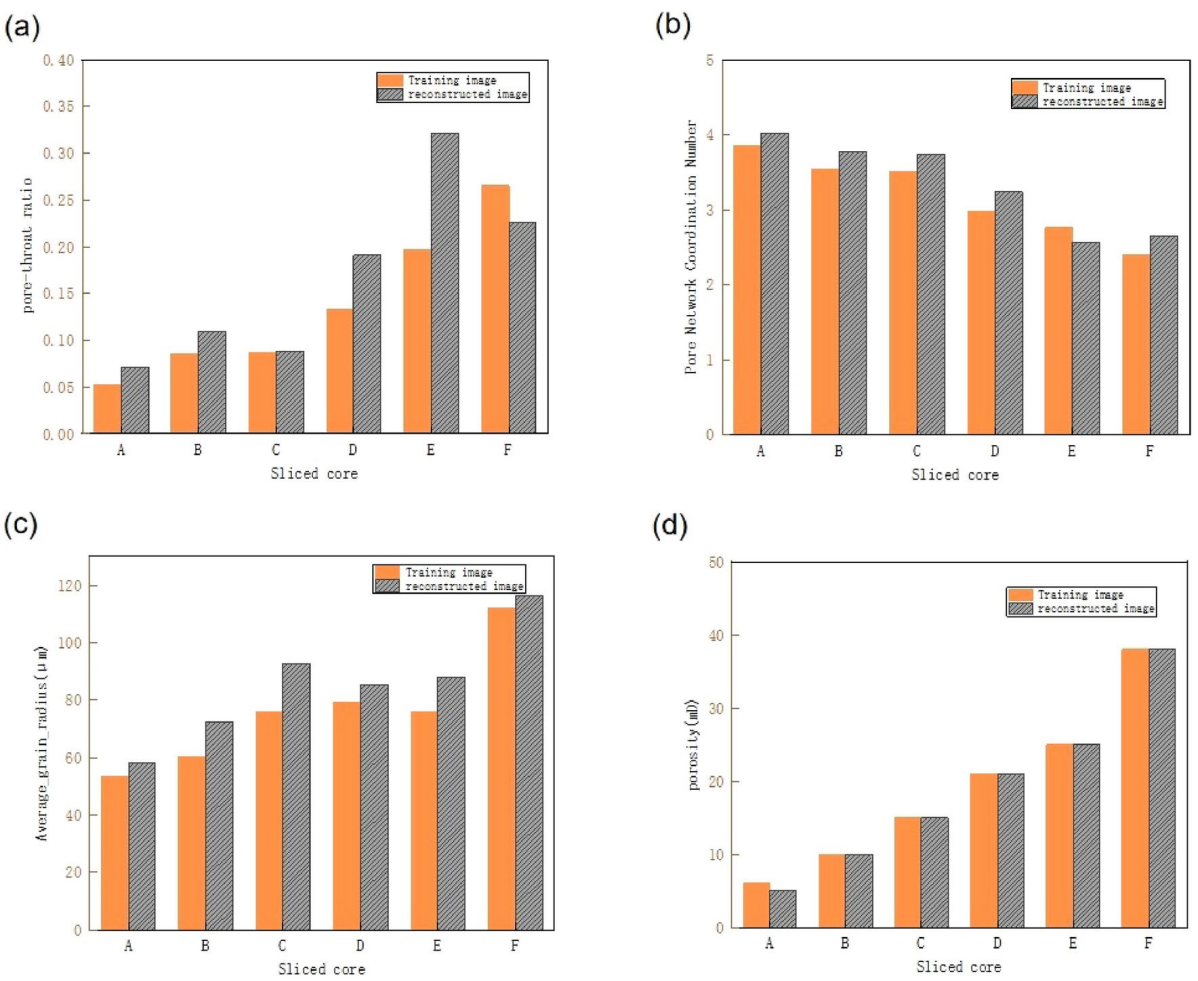
(**a**) Comparison of pore-throat ratios between TI and RI for different slices. (**b**) Comparison of coordination numbers between TI and RI for different slices. (**c**) Comparison of average particle size radii between TI and RI for different slices. (**d**) Comparison of porosity between TI and RI for different slices.

The pore-throat ratio, which is the ratio of pore diameter to throat diameter, is an important parameter for characterizing pore structure and a crucial microphysical property of reservoir media. A larger pore-throat ratio indicates a larger pore space and wider channels relative to the throats, which can be more favorable for fluid flow. Figure 8a displays the pore-throat ratios of the pore structures in different core slices, with yellow bars representing the TI and gray bars representing the RI. In most slice samples, the pore-throat ratios of the RI maintain the same trend as those of the TI, indicating that the reconstruction method effectively reproduces the pore structure features of the TI overall. Notably, in sample C, the pore-throat ratio of the RI closely matches the pore structure features of the TI. In some samples, such as sample E, the pore-throat ratio in the RI is significantly higher than in the TI. This discrepancy may be due to the reconstruction method’s inability to fully capture the features of complex pore structures, reflecting limitations in detail handling and accuracy. However, overall, the RI for all six samples follow the same trend as the TI, demonstrating that the reconstruction method has high applicability and reliability.

Figure 8b uses the average coordination number to quantitatively assess the connectivity of pore structures in porous media. the coordination number refers to the number of nearest neighboring particles surrounding a given particle, describing the degree of connectivity of particles in space. A higher coordination number indicates better pore connectivity. Figure 8b displays histograms of the average coordination number distribution for core slices corresponding to TI and RI. In the figure, yellow represents the coordination numbers of the pore structures in the initial TI, while gray represents the coordination numbers in the RI. For the six samples, which are from the same core slice, the pore coordination numbers are concentrated in the range of 2–4. For samples with the same porosity, the pore coordination numbers in the RI are generally consistent with those in the TI. As porosity increases, the average coordination number for the six samples with different porosities gradually decreases. This indicates that, at lower porosities, the connectivity of pores at the microscopic scale is better.

The grain size radius of pores is a critical parameter affecting pore structure and directly relates to the fluid flow characteristics of the pores. Pores with larger grain size radii typically provide larger fluid flow channels, while pores with smaller grain size radii present greater flow resistance. Figure 8c shows the average grain size radius for TI and RI under different core slices, with yellow representing the TI and gray representing the RI.It is observed that the grain size radius of the RI is similar to that of the TI for most samples, although some differences exist. In certain samples, the grain size radius in the RI closely matches that of the TI, indicating that the reconstruction method performs well for these samples and can effectively reproduce the pore structure of the TI. However, in some samples, there are noticeable differences in the grain size radius between the RI and the TI, which may be due to errors or limitations in the reconstruction algorithm for these samples. Overall, the trend of the grain size radius in the RI is consistent with that of the TI, suggesting that the reconstruction method retains the pore structure characteristics of the TI to a certain extent. For specific samples with significant discrepancies in grain size radius, further optimization of the reconstruction algorithm may be needed to improve accuracy.

Porosity is a key parameter describing the size and distribution of pore space and has a significant impact on the fluid flow characteristics of pores. Higher porosity typically indicates larger pore space and lower fluid flow resistance, while lower porosity suggests smaller pore space and higher fluid flow resistance. Figure 8d shows the porosity of the initial and reconstructed two-dimensional pore images for six core slice samples. Analyzing the porosity of the TI and RI is the most direct way to assess reconstruction quality. The figure reveals that, except for the sample with 6% porosity, which shows a very slight difference in porosity between the TI and RI, the porosity of the RI for the other samples is consistent with that of the TI. This indicates that the reconstruction method performs well for these samples and can effectively reproduce the pore structure of the TI. Although some samples show slightly higher or lower porosity in the RI, which could affect the fluid flow characteristics of the pore structure, overall, the reconstruction method demonstrates performance in retaining the initial pore structure.

The variogram describes the spatial correlation between two points by measuring the difference in values between them. Typically, the definition of the variogram is as follows $\gamma \left( h \right) = \left[ {Z\left( x \right) - Z\left( {x + h} \right)} \right]^{2}$,In this context,$\gamma \left( h \right)$ is the variogram, representing the variability between two points at a distance of $h$.$Z\left( x \right)$ and $Z\left( {x + h} \right)$ represent the values at locations $x$ and $x + h$, respectively.$h$ is the distance, typically indicating the spatial interval between the two points.

**Fig. 9:**
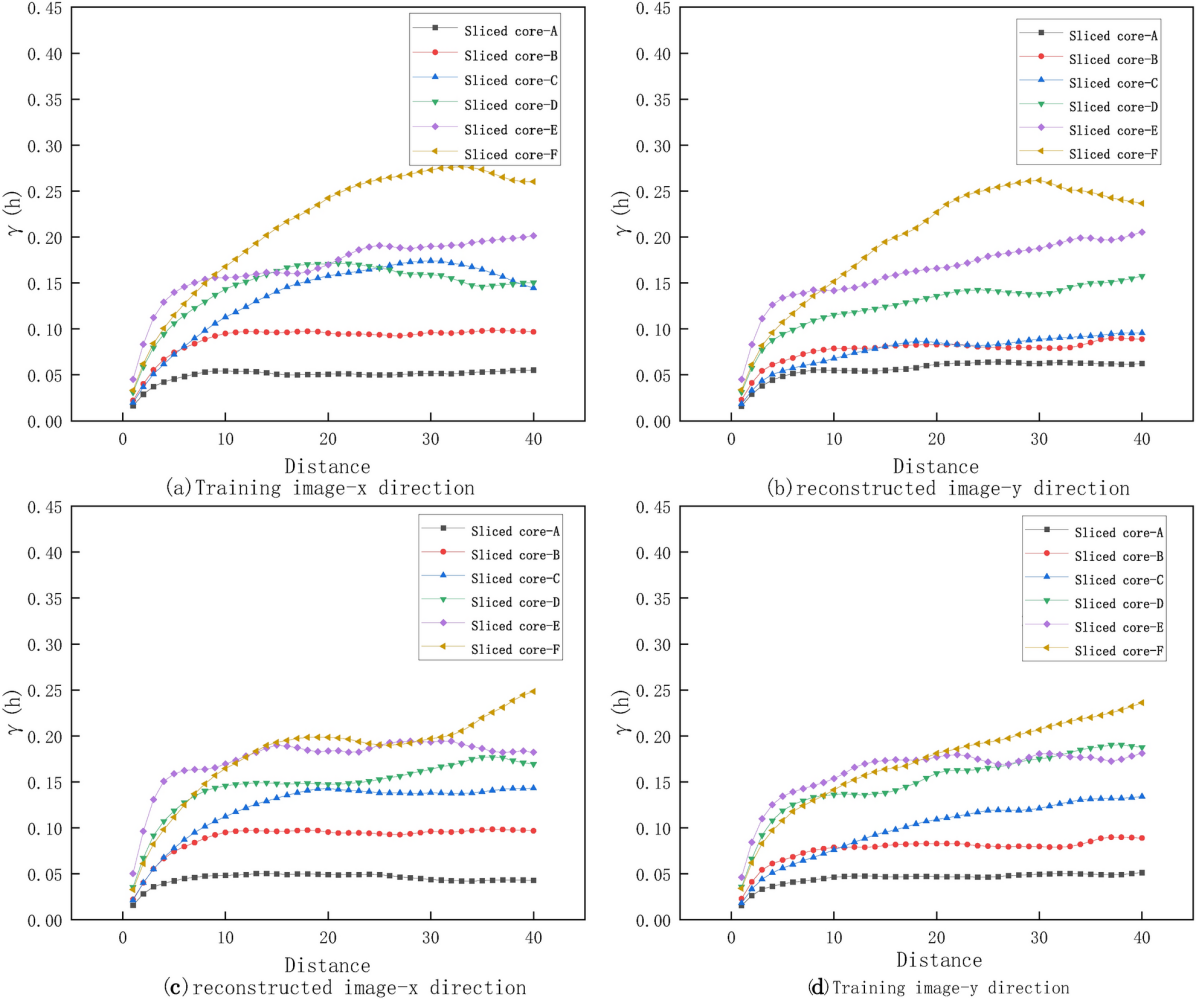
Variogram analysis of TI and RI: seven color-coded lines represent different slices, with lag distance on the X-axis and variogram value on the Y-axis. (**a**) Variogram of the TI in the x-direction. (**b**) Variogram of the TI in the y-direction. (**c**) Variogram of the RI in the x-direction. (**d**) Variogram of the RI in the y-direction.

Figure 9 compares the differences between the TI and RI from the x and y directions in two-dimensional images to analyze the reconstruction effects. Observing the TI (top left and top right), the variance function for different core slices increases with lag distance, indicating that the pore structure has a certain spatial correlation at larger scales. The trends in the variance function in the x and y directions are generally consistent across different slices, showing similar spatial variability in these directions. For the RI (bottom left and bottom right), the variance function also increases with lag distance for different core slices, but exhibits some fluctuations at certain lag distances. While the variance function trends in the x and y directions for the RI show some similarity to the TI, there are differences at larger lag distances. Comparing the variance functions of the TI and RI, it is evident that there are significant differences in the variance function values at certain lag distances in the RI compared to the TI. This may be due to the loss of certain detail information during the reconstruction process or limitations in the reconstruction method when dealing with variability at specific scales. However, the overall trend of the variance function across different core slices is consistent between the TI and RI, indicating that the reconstruction method retains the spatial structural features of the TI to some extent. summary, the reconstruction method performs well in reproducing the spatial variability of the TI at smaller lag distances but may need further improvement at larger lag distances to better capture the spatial correlations of the TI.

**Fig. 10:**
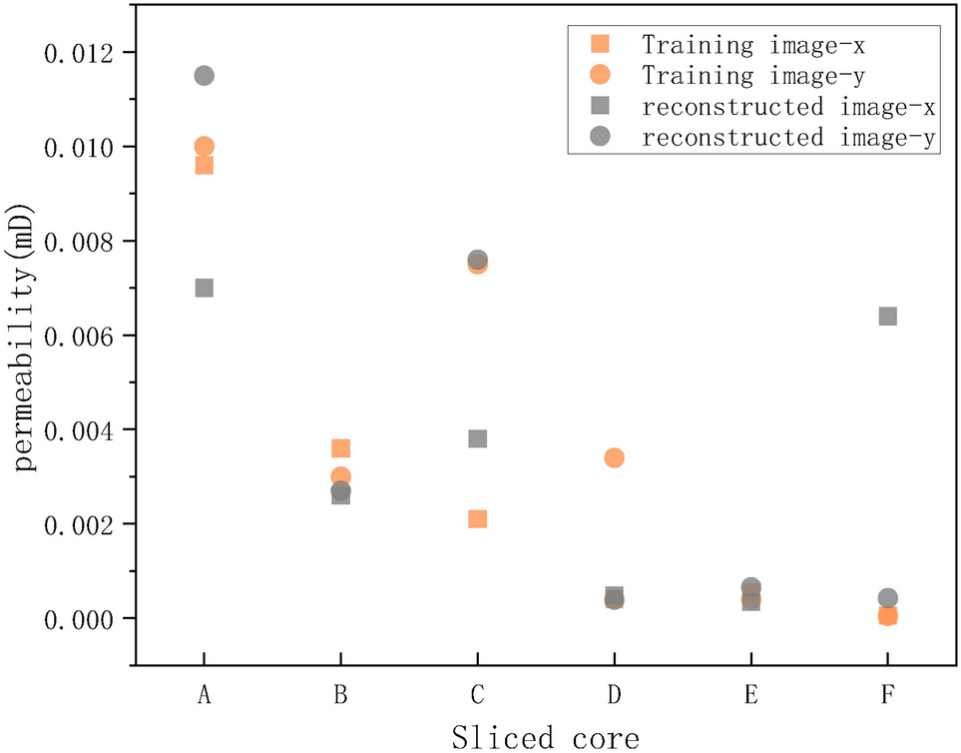
Comparison of permeability between TI and RI of pore slices in different porous media.

The pore structure directly affects the permeability characteristics of porous media. Permeability is a physical property that measures the ability of a material to allow fluids to pass through, commonly used to characterize the fluid transport properties of porous media. Higher permeability indicates more connected pore spaces and smoother fluid flow. By observing the permeability of different samples in the figure, it can be inferred that the RI have effectively retained the connectivity of the original pore structure in certain samples, resulting in higher permeability. Figure 10 displays the permeability distribution for six core slice samples, where yellow squares and yellow balls represent the permeability data of the TI along the x and y axes, respectively, and the gray squares and gray balls represent the permeability data of the RI along the x and y axes. In some samples (e.g., samples B and E), the permeability of the RI is close to that of the TI, indicating that the reconstruction method effectively reproduces the permeability characteristics of the original images for these slices. However, in other samples (e.g., samples C and F), there are some differences in permeability between the RI and TI, which may reflect the limitations of the reconstruction method in certain situations. Overall, the reconstruction method performs excellently in preserving the overall connectivity and permeability of the pore structure. However, there may be a need for further optimization and improvement when dealing with complex or highly heterogeneous pore structures.

The SNESIM (Sequential Nearest Neighbor Simulation) algorithm generates new synthetic images using a TI to reconstruct pore structures, aiming to preserve the spatial patterns and statistical properties of the original image. Through experimental analysis, the main differences between the RI and the TI are as follows:

1. Structural Differences: While the RI retains the overall spatial correlation of the TI, there may be differences in fine details, mainly manifested as small-scale variations.
2. Image Continuity: Although the TI may have certain discontinuities or irregularities, SNESIM improves the continuity of the RI by ensuring statistical consistency between neighboring pixels.
3. Pattern Randomization: The inherent randomness of SNESIM leads to differences in specific details between the RI and the TI. The algorithm focuses on matching large-scale patterns (such as local heterogeneity and directional trends) and may not replicate every minute detail.

Nevertheless, the overall experimental results show that the RI generated at different scales maintain good consistency in porosity with the TI. Average pore diameter, permeability, and other pore structure indicators. These differences are generally within an acceptable range, meaning that the RI effectively retains the essential characteristics of the TI and can be used for further analysis.

## Conclusion

Based on the comprehensive analysis of the experiments, the SNESIM algorithm demonstrates good performance in reconstructing pore structures. The analysis of parameters such as porosity, pore-throat ratio, average particle radius, coordination number, and permeability shows that the RI generally maintain trends similar to those of the TI in most samples. The performance parameters of the reconstructed core slices are largely consistent with those of the initial core slices, indicating that the SNESIM algorithm has good applicability and reliability in pore structure reconstruction, effectively reproducing the main pore structure features of the initial TI.

However, discrepancies still exist between the RI and the TI. Insufficient and unevenly distributed conditional data can lead to incomplete recovery of certain details. Additionally, the randomness inherent in filling grid nodes introduces variations in the microstructure. Improper settings of algorithm parameters, such as template size and search window, can also result in the loss of details. Furthermore, it is challenging to fully retain multi-scale structures during reconstruction, especially when it comes to large-scale structures and connectivity, which are prone to deviations. Edge effects may also occur, leading to lower reconstruction accuracy at the image boundaries. By optimizing parameter settings, increasing the diversity of conditional data, and improving multi-scale methods, these differences can be mitigated to some extent, resulting in RI that better match the original structure. However, the analysis of pore performance parameters in this study is not comprehensive. Parameters such as tortuosity and constrictivity, which are crucial for evaluating pore reconstruction effects, have not been fully considered. Moreover, the core slices used in this study were all obtained from the same core, which somewhat limits the comprehensive understanding of pore structures. Effectively transferring the pore structures from the 2D plane to a 3D pore space, while maximizing the preservation of these structures, still requires further analysis and research. Specifically, when dealing with complex pore structures, the accuracy and performance of reconstruction algorithms need further improvement. Future research should focus on optimizing the algorithm to handle a wider variety of pore structures. Additionally, the evaluation of pore reconstruction performance should not be limited to parameters like porosity and pore diameter; a more comprehensive analysis should include parameters that affect the effective transport properties of porous media, such as pore structure tortuosity, constrictivity, and Euler number. Moreover, efforts should be made to explore 3D reconstruction methods that provide a more complete description and analysis of pore characteristics in real porous media.

Although SNESIM algorithm has better reconstruction accuracy, its efficiency is limited by factors such as database size, search template complexity, sample point selection, and computational load. To improve efficiency, strategies include optimizing template selection and narrowing the search window to reduce database requirements. Utilizing a multi-grid approach, which enhances computational efficiency through coarse simulations followed by gradual refinements, is also effective. Additionally, employing parallel computing and GPU acceleration can significantly reduce computation time. Introducing intelligent sample selection algorithms can minimize redundant modeling and improve modeling quality. Dynamic threshold adjustment and parameter optimization can make the model more adaptable to different image features, further enhancing reconstruction performance and efficiency. These improvements can effectively increase the practicality and computational efficiency of the SNESIM algorithm.

## Supplementary Information

Supplementary Information.

## Data Availability

The datasets used and/or analysed during the current study available from the corresponding author on reasonable request.
