# Supplementary material for: Reconstruction of porous media pore structure and simulation effect analysis of multi-index based on SNESIM algorithm
Source: Sci Rep. 2025 Feb 10;15:4856. doi: 10.1038/s41598-025-88730-w (PMC11808111; doi:10.1038/s41598-025-88730-w)
Supplement: Supplementary file 1 — Supplementary Information. [file 41598_2025_88730_MOESM1_ESM.docx]

| symbol | | Description |
| --- | --- | --- |
|  | | Spatial structure variables on the domain of the trained image |
| $d(u)_{n}$ | $u$ center location | data event |
|  | $n$ state value of size n |  |
| $\tau_{\text{n}}$ | | data template |
| $c\left(d\left(\text{n}\right)\right)$ | | the repetition count of $d(n)$for the data event |
| $\gamma\left(h\right)=\left[Z\left(x\right)-Z\left(x+h\right)\right]^{2}$ | | $\gamma\left(h\right)$ variogram |
|  |  | $Z\left(x\right)$ the values at locations$x$ |
|  |  | $Z\left(x+h\right)$ the values at locations$x+h$ |
